# Adjuvant interferon gamma in patients with pulmonary atypical Mycobacteriosis: A randomized, double-blind, placebo-controlled study

**DOI:** 10.1186/1471-2334-8-17

**Published:** 2008-02-11

**Authors:** María T Milanés-Virelles, Idrian García-García, Yamilet Santos-Herrera, Magalys Valdés-Quintana, Carmen M Valenzuela-Silva, Gaspar Jiménez-Madrigal, Thelvia I Ramos-Gómez, Iraldo Bello-Rivero, Norma Fernández-Olivera, Reinaldo B Sánchez-de la Osa, Carmen Rodríguez-Acosta, Lidia González-Méndez, Gregorio Martínez-Sánchez, Pedro A López-Saura, the MACGAM Study Group

**Affiliations:** 1 "Benéfico Jurídico" Hospital, Havana, Cuba; 2 Center for Biological Research, Clinical Trials Division, Havana, Cuba; 3 "Amalia Simoni" Hospital, Camagüey, Cuba; 4 University of Havana, Institute of Pharmacy and Food, Havana, Cuba

## Abstract

**Background:**

High antibiotic resistance is described in atypical Mycobacteriosis, mainly by *Mycobacterium avium *complex (MAC).

**Methods:**

A randomized, double-blind, placebo-controlled clinical trial was carried out in two hospitals to evaluate the effect of interferon (IFN) gamma as immunoadjuvant to chemotherapy on patients with atypical mycobacteria lung disease. Patients received placebo or 1 × 10^6 ^IU recombinant human IFN gamma intramuscularly, daily for one month and then three times per week up to 6 months as adjuvant to daily oral azithromycin, ciprofloxacin, ethambutol and rifampin. Sputum samples collection for direct smear observation and culture as well as clinical and thorax radiography assessments were done during treatment and one year after. Cytokines and oxidative stress determinations were carried out in peripheral blood before and after treatment.

**Results:**

Eighteen patients were included in the IFN group and 14 received placebo. Groups were homogeneous at entry; average age was 60 years, 75% men, 84% white; MAC infection prevailed (94%). At the end of treatment, 72% of patients treated with IFN gamma were evaluated as complete responders, but only 36% in the placebo group. The difference was maintained during follow-up. A more rapid complete response was obtained in the IFN group (5 months before), with a significantly earlier improvement in respiratory symptoms and pulmonary lesions reduction. Disease-related deaths were 35.7% of the patients in the placebo group and only 11.1% in the IFN group. Three patients in the IFN group normalized their globular sedimentation rate values. Although differences in bacteriology were not significant during the treatment period, some patients in the placebo group converted again to positive during follow-up. Significant increments in serum TGF-beta and advanced oxidation protein products were observed in the placebo group but not among IFN receiving patients. Treatments were well tolerated. Flu-like symptoms predominated in the IFN gamma group. No severe events were recorded.

**Conclusion:**

These data suggest that IFN gamma is useful and well tolerated as adjuvant therapy in patients with pulmonary atypical Mycobacteriosis, predominantly MAC. Further wider clinical trials are encouraged.

**Trial registration:**

Current Controlled Trials ISRCTN70900209.

## Background

The prevalence of isolated atypical Mycobacteria (also called non-tuberculous Mycobacteria) infection is growing and is suspected to surpass *Mycobacterium tuberculosis *in some areas. *Mycobacterium avium *complex (MAC) species are the most frequent. Immunocompetent patients develop lung disease as a progressive chronic pneumonia. In the United States and in Japan there are approximately 1.3 cases per 100 000 persons, whereas in Switzerland, there are 0.9 cases per 100 000 persons with pulmonary MAC. It is postulated that up to 70% of patients with pulmonary MAC infection had a previous bronchopulmonary disorder [1]. However, cases without a predisposed previous condition or identifiable immunodeficiency also exist [2].

Atypical Mycobacteria can survive and proliferate within the hostile environment of host macrophages [3,4]. The treatment of these pulmonary infections is more difficult than *Mycobacterium tuberculosis *due to a higher drug resistance. MAC and *Mycobacterium fortuitum-cheloneae *are the two most resistant. The problem to choose an adequate antibiotic combination derives from the lack of controlled, prospective studies. The different populations, bacteriological methods and cure approaches applied, as well as lack of correlation between clinical and laboratory results add higher complexity. Conventional chemotherapy is prolonged and associated to frequent toxicities [5-7]. Mortality attributed directly to infection occurs, but its rate is ignored.

Therefore, an alternative therapeutic target can be directed to the manipulation of the host's defenses. As most of the intracellular infections, the immune response to control infection depends primarily on a Th1 lymphocytes outbreak [8]. Interferon (IFN) gamma, a glycoprotein produced primary by CD4+ cells, plays a main role in this process. Enough evidences exist related to the action of IFN gamma on the immunoregulatory activity of macrophages [9]. Lack of production of this cytokine [10] or expression of its receptor [11-13] is associated to the infection's most lethal forms. IFN gamma has also a potent antifibrotic effect [14,15].

Previous clinical studies have demonstrated satisfactory results with the use of recombinant IFN gamma in patients infected with atypical Mycobacteria [16-20]. However, none of these reports was a controlled trial and they included only few patients. The present placebo-controlled study was done with the objective to assess the immunoadjuvant IFN gamma effect in patients with pulmonary atypical Mycobacteriosis regarding their clinical, bacteriological and radiological evolutions. Additionally, several immune response and oxidative stress markers were measured. The safety of the procedure was also evaluated.

## Methods

A randomized, double-blind, placebo-controlled trial was carried out at the "Benéfico Jurídico" Hospital, Havana, which is the national reference unit for tuberculosis and other respiratory diseases and where most of the patients with unfavorable evolution are remitted. Another participant institution was the "Amalia Simoni" Hospital, Camagüey. The clinical protocol was approved by the Ethics Committees of the corresponding sites and by the Cuban Regulatory Authority. The study complied with the Declaration of Helsinki.

### Patients

The study population was constituted by Cuban patients, more than 18 years-old with diagnosis of pulmonary atypical Mycobacteriosis, who gave their written, informed consent to participate. The diagnosis comprised isolation and classification of any of the atypical *Mycobacteria *species three or more times in sputum-culture samples, symptoms such as cough and expectoration, and tuberculosis-like pulmonary lesions at thorax radiography. Exclusion criteria were other concomitant pulmonary infections, chronic diseases or neoplasia, pregnancy or nursing, severe psychiatric dysfunction, multiple sclerosis or any other autoimmune disorder, HIV co-infection, hypersensitivity to IFN gamma or any other drug used in the trial, and treatment with corticosteroids or other immunosuppressor medication. Patients that had received any type of IFN during the 3 previous months were also excluded.

### Study design and treatment

Patients were distributed according to a computer-generated random number list, stratified by center, to receive intramuscular IFN gamma as adjuvant to oral chemotherapy (IFN group) or chemotherapy plus placebo (placebo group) during 6 months. The study was double-blinded. IFN gamma and placebo vials looked the same, labeled with the hospital code and the patient's inclusion number. Their composition differed only regarding the presence of IFN. Random lists were kept at the sponsor's product-preparation department, where vials were labeled. This group was independent from the monitors, investigators or other trial participants. All clinical and laboratory evaluations were done blindly regarding the patients' group allocation. The code was opened only in case of serious adverse events, deaths or at the end of the study, after databases closure.

Treatment schedule consisted in 1 × 10^6 ^IU of human recombinant IFN gamma (produced in *E. coli*, specific activity: 1 × 10^7 ^IU/mg of proteins; Heberon Gamma R^®^, Heber Biotec, Havana) or placebo intramuscularly, daily during 4 weeks and then 3 times per week for the next 20 weeks. All the patients received the same conventional antibiotic schedule. Drugs were given orally, daily as follows: azithromycin 500 mg, ciprofloxacin 1000 mg, rifampin 600 mg, and ethambutol 2000 mg.

Subjects were hospitalized during the first 4 weeks of treatment to check the initial evolution and for a better assessment of immediate adverse events. Antipyretic medication was given orally at the same time as the first IFN injections, in order to mitigate the expected IFN-dependent flu-like syndrome and keep the blinding. Afterwards, patients were followed up to 18 months as out-patients. Non-responders or relapsers continued under antibiotic therapy during this period.

### Evaluation

The main efficacy outcome was an overall response that integrated clinical, bacteriological and radiological results, at the end of treatment (month 6) and after 12 additional months of follow-up (month 18). This composite variable was considered as complete if all symptoms disappeared, sputum acid-fast-bacilli smear and culture were negative, and X-ray pulmonary lesions improved. Partial response included symptoms decrease, negative sputum smear and culture, and improvement or stabilization at X-ray. Symptoms persistence, positive bacteriological examinations, or X-ray lesion progression was considered as no response. The clinical, radiological and bacteriological evaluations were also taken independently as secondary variables, as well as time to response and overall survival.

The reduction or disappearance of the respiratory symptoms was the first criteria to consider clinical improvement. Physical examination of the respiratory tract, bodyweight, vital signs and any other particular characteristic were taken into account. General clinical status was classified as good, moderate or bad. Good: none or discreet respiratory symptoms without frequent or serious exacerbations, appropriate bodyweight; moderate: maintained respiratory symptoms with more acute exacerbations, appropriate bodyweight; bad: maintained serious respiratory symptoms (intense dyspnea, cough, and abundant expectoration), low bodyweight. General clinical status improvement was considered when the patient passed from "bad" to "moderate" or from "moderate" to "good".

Sputum samples were taken for acid-fast-bacilli smear and culture. Regular specimen staining methods (Zielh-Neelsen) and culture (Lowenstein-Jensen medium) were used. Atypical *Mycobacteria *species were identified by characteristic colony morphology, growth rate, and pigmentation (the Runyon classification system), using a battery of routine chemical reactions. Codification was defined as the number of bacilli counted in 300 fields on the direct observation or the number of colonies grown in the culture as follows: code 0: no bacilli or colonies; code 1 to 5: the same number of bacilli counted or colonies grown; code 6: 6 – 24 bacilli or colonies; code 7: 25 – 100 bacilli or colonies; code 8: more than 100 bacilli or colonies; code 9: bacilli in most fields counted or continuous growth.

Pulmonary lesions extension at radiography was considered minimum if they comprised up to one third of a lung area; moderate up to one lung involvement, and advanced if more extensive. Radiological worsening was defined by the presence of new infiltrates, cavitations, multiple nodules or increase of the fibrosis area. Stabilization was considered when new lesions did not appear and the extension of fibrosis-derived damage did not increase. Lesion extension reduction together with respiratory symptoms decrease was taken as improvement.

Safety and tolerability were monitored by means of a rigorous adverse events control. Additionally, blood samples were taken for routine hematological and biochemical determinations.

### Laboratory procedures

Gene modulation was studied in both groups before and after the 6 months treatment using a RT-PCR for mRNA expression. Briefly, peripheral blood mononuclear cells (PBMC) were separated from heparinized whole blood (10–15 mL) by Ficoll-Paque plus (Amersham Biosciencies, endotoxin tested) gradient centrifugation and RNA was extracted using the acid guanidium thiocyanate-phenol-chloroform method. RNA was then reverse transcribed using the Perkin Elmer core kit (Perkin-Elmer/CETUR, Norwalk, CT). Samples were incubated 10 min at room temperature and then 15 min at 42°C and 5 min at 99°C. Primers used were: 5'-CCA TGG AGA AGG CTG GGG-3' and 5'-CAA AGT TGT CAT GGA TGA CC-3' for glyceraldehyde-3-phosphate dehydrogenase (GAPDH), 5'-ATG AAA TAT ACA AGT TAT ATC TTG GCT TT-3' and 5'-GAT GCT CTT CGA CCT CGA AAC AGC AT-3' for IFN gamma, 5'-TGC CAG GCA GGT TCT CTT CC CG-3' and 5'-GGT TAT CTC TCA GCT CCA CGC CA-3' for tumor necrosis factor (TNF) alpha, 5'-CAA GCA GAG TAC ACA CAG CA-3' and 5'-GAT GCT GGG CCC TCT CAA GC-3' for transforming growth factor (TGF) beta, 5'-AAC TGA AGC TCG CAC TCT CG-3' and 5'-TCA GCA CAG ATC TCC TTG GC-3' for monocyte chemoattractant protein (MCP)-1, 5'-AAG GTG GCA GGA TGT CTC GTG-3' and 5'-TGG TCT CGT GTT CTT CTG TTC TG-3' for signal transducer and activator of transcription (STAT)-1, 5'-AAC AAG ACC CAG ACA TCA AG-3' and 5'-GAG GTA CAA TAA GGT TTC TCA AG-3' for interleukin (IL)-10 and 5'-GAT GTT TGT GGA CGT GGT CTT G-3' and 5'-GAT GTT TGT GGA CGT GGT CTT G-3' for transcription factor associated to Th1 lymphocytes (T-bet). Amplification was done in an automatic thermal cycler (Eppendorff) at 94°C for 30 sec, 56°C for 30 sec. and 72°C for 30 sec. After 35 cycles, PCR amplification products were resolved following separation by 2% agarose gel electrophoresis. The bands were visualized by ethydiumbromide staining and then quantified by gel densitometric scanning (Molecular Analyser software for Windows, version 1.4.1). The ratios between target cDNA and GAPDH as housekeeping gene were evaluated and expressed as relative amount of genetic expression.

Oxidative stress parameters were also measured in serum before and after treatment using an ultramicroanalitic system and the Ultrospect Bonus Spectrophotomer (Pharmacia LKB). Total superoxide dismutase (SOD) activity was determined using the pyrogallol autoxidation method. Catalase activity was evaluated following hydrogen peroxide decomposition [21]. Lipid peroxidation was measured through malonyldialdehyde (MDA) concentration [22]. Potential lipid peroxidation products (PLPP) [23], total hydroperoxides (THP) [24] and advanced oxidation protein products (AOPP) [25] were also measured, as recommended.

Hematological counts and blood chemistry were done according to usual clinical laboratory procedures, using advanced automated analyzers. These included hemoglobin, hematocrit, globular sedimentation rate (GSR), leukocytes and platelets counts, transaminases, bilirubin, creatinine and urea. All laboratory analyses were done blindly.

### Statistics

Sample size was calculated using the log-rank test ("2N Program for Design of Clinical Trials") assuming a 30% overall response in the control group and 90% in the IFN group, with 0.05 and 0.8 for types I and II errors, respectively, at six months of evaluation. A 20% excess was considered to compensate withdrawals. This yielded a total of 34 patients for the trial.

Data were double entered and validated on Microsoft Access and then imported into SPSS version 13.0 for further analysis. Continuous variables were expressed as mean ± standard deviation (SD) or median ± interquartile range (QR) and minimum and maximum values (range). With these variables a normality analysis (Shapiro Wilk's test) and homogeneity of variance (Levene's test) were carried out. Categorical variables were given as frequencies and percentages. Times to response and survival were expressed as mean ± standard error (SE) and their 95% confidence interval. Groups were compared using the Fisher's exact or chi-square tests for all categorical variables and the Student's t-test (parametrical) or Mann-Whitney's U test (non-parametrical) for continuous variables. The log-rank test was used to compare times to response and survival. Kaplan-Meier survival curves were also estimated. Significance level chosen was 0.05. Analyses of the overall response and clinical outcome were done under "intention-to-treat" basis, where missing data were considered as failures. Stratified analyses according to investigation site were not possible due to the small sample size in one of them (see below).

## Results

Thirty-two patients were enrolled from October 2002 to March 2005; then follow-up continued up to September 2005. Eighteen patients received IFN gamma and 14 placebo. Most of the patients (84.4%) corresponded to "Benéfico Jurídico" Hospital. Table 1 summarizes the course of the trial. Sixteen patients (88.9%) completed the 6-months treatment period in the IFN group (two abandoned after the first month), while 10 (71.4%) completed it in the placebo group. Withdrawals in this latter group were 2 deaths after the first month (MAC infection and pulmonary thromboembolism) and 2 voluntary abandons after months 2 and 5. The patient that abandoned after the second month of treatment died 2 months later due to a lung cancer, not present at entry or at withdrawal. During follow-up 2 patients died in the placebo group (myocardial infarction and pulmonary thromboembolism) and other 2 in the IFN group (both myocardial infarctions). In addition, one IFN patient did not attend the last evaluation. Finally, 13 patients (72.2%) completed the entire study in the IFN group, and 8 (57.1%) in the placebo group.

**Table 1:** Course of the trial.

	**IFN gamma**	**Placebo**
Randomized patients	18	14
Treatment interruptions	2 (11.1%)	4 (28.6%)
Voluntary abandoners	2 (11.1%)	2 (14.3%)
Deaths	0	2 (14.3%)
Completed 6 months of treatment	16 (88.9%)	10 (71.4%)
Follow-up withdrawals	3 (16.7%)	2 (14.3%)
Voluntary abandoners	1 (5.5%)	0
Deaths	2 (11.1%)	2 (14.3%)
Completed 12 months of follow-up	13 (72.2%)	8 (57.1%)
Total deaths	2 (11.1%)	5* (35.7%)

* One of the voluntary abandoners in this group died

Baseline characteristics of the included patients are shown in Tables 2 and 3. Groups were homogeneous. Even if there were noteworthy differences concerning smoking and alcohol drinking habits, the sample size does not provide enough power to assess their relation to response. Mean age was around 60 years for both groups and white men prevailed (> 70%). *M. avium-intracellulare *complex was 94% of the isolated species. None of the respiratory antecedents stood out particularly. All the patients presented breathing disorders (movements, crepitations) at physical examination, but none had any extra-pulmonary manifestation of the disease.

**Table 2:** Characteristics of the study population at entry.

**Characteristic**	**IFN gamma N = 18**	**Placebo N = 14**
Age (yrs), median ± IQR	62 ± 11	57 ± 18
Male gender	13 (72.2%)	11 (78.6%)
White	16 (88.9%)	11 (78.6%)
BMI (Kg/m^2^), mean ± SD	21.1 ± 3.5	18.7 ± 2.8

Toxic habits		
Smokers	5 (27.8%)	7 (50.0%)
Alcohol	1 (5.6%)	5 (35.7%)

Organism		
*M. avium *complex	17 (94.4%)	13 (92.9%)
*M. fortuitum-cheloneae*	1 (5.6%)	0
*M. kansasii*	0	1 (7.1%)

Months since diagnosis, median ± IQR	30 ± 40	40 ± 34

Respiratory tract history		
Asthma	0	2 (14.3%)
Tuberculosis	2 (11.1%)	0
Minimum pleurotomy	2 (11.1%)	0
Right superior lobectomy	0	1 (7.1%)
Emphysematous bullous disease	1 (5.6%)	0
Fibroemphysema	0	1 (7.1%)
Interstitial pulmonary fibrosis	1 (5.6%)	0
Pneumothorax	1 (5.6%)	0

BMI: Body mass index.

**Table 3:** Clinical, radiological, bacteriological and overall outcomes during the trial.

**Evaluation**	**Month**		**IFN gamma**	**Placebo**	**P (test)**
**Overall response**					

Responders ^(a) ^(intention-to- treat)	6		13/18 (72.2%)	5/14 (35.7%)	0.037 (χ^2^)
	18		12/18 (66.7%)	4/14 (28.6%)	0.030 (χ^2^)
Responders (last evaluation)			15/18 (83.3%)	5/14 (35.7%)	0.005 (χ^2^)

**Clinical**					

Dyspnea	0		15/18 (83.3%)	13/14 (92.9%)	
	6		1/15 (6.7%)	3/9 (33.3%)	0.27 (FE)
	18		1/13 (7.7%)	3/8 (37.5%)	0.25 (FE)

BMI (kg/m^2^), mean ± SD	0		21.1 ± 3.5 (N = 18)	18.7 ± 2.8 (N = 14)	
	6		21.8 ± 3.7 (N = 14)	19.4 ± 3.0 (N = 9)	0.88 (St)
	18		22.9 ± 4.4 (N = 13)	19.9 ± 3.9 (N = 8)	0.15 (St)

Good general status (intention-to-treat)	0		3/18 (16.7%)	4/14 (28.6%)	
	6		13/18 (72.2%)	5/14 (35.7%)	0.037 (χ^2^)
	18		12/18 (66.7%)	4/14 (28.6%)	0.03 (χ^2^)

Improvement ^(b) ^(intention-to-treat)	6		13/18 (72.2%)	5/14 (35.7%)	0.037 (χ^2^)
	18		12/18 (66.7%)	4/14 (28.6%)	0.03 (χ^2^)

**Radiological**					

Lesion extension	0	Adv	12 (66.7%)	11(78.6%)	
		Mod	5 (27.8%)	3 (21.4%)	
		Min	1 (5.6%)	0	
	
	6	Adv	2 (13.3%)	5 (55.6%)	1.00^c ^(FE)
		Mod	12 (80.0%)	3 (33.3%)	
		Min	1 (6.7%)	1 (11.1%)	
	
	18	Adv	1 (7.7%)	2 (25.0%)	0.085^c ^(FE)
		Mod	5 (38.5%)	5 (62.5%)	
		Min	7 (53.8%)^d^	1 (12.5%)	

Improvement (intention to treat)	6		12/18 (66.7%)	6/14 (42.8%)	0.32 (χ^2^)
	18		13/18 (72.2%)	4/14 (28.6%)	0.036 (χ^2^)

Cavitary lesions disappearance			5/12 (41.7%)	1/12 (8.3%)	0.15 (FE)

**Bacteriological**					

Sputum- Direct (+)	0		14/18 (77.8%)	10/14 (71.4%)	
	Cod.		7 ± 4	8 ± 8	
	
	6		1/15 (6.7%)	2/10 (20.0%)	0.54 (FE)
	Cod.		0 ± 0	0 ± 2	0.28 (MW)
	
	18		1/13 (7.7%)	3/8 (37.5%)	0.253 (FE)
	Cod.		0 ± 0	0 ± 7	0.112 (MW)
	
	Relapse		1/13 (7.7%)	3/8 (37.5%)	0.25 (FE)

Sputum- Culture (+)	0		18 (100%)	14 (100%)	
	Cod.		8 ± 2	9 ± 4	
	
	6		2/15 (13.3%)	2/10 (20.0%)	1.00 (FE)
	Cod.		0 ± 0	0 ± 2	0.60 (MW)
	
	18		1/13 (7.7%)	4/8 (50.0%)	0.11 (FE)
	Cod.		0 ± 0	0 ± 8	0.042 (MW)
	
	Relapse		1/13 (7.7%)	3/8 (37.5%)	0.25 (FE)

(St): Student's t test; (MW): Mann-Whitney's U test; all binary variable comparisons were with the Fisher's exact test.

^(a) ^All overall responses were complete except for one IFN group case at month 6 with partial response.

^(b) ^General clinical status improvement if the patient passed from "bad" to "moderate" or from "moderate" to "good".

Adv: Advanced; Mod: Moderate; Min: Minimum; ^(c) ^Combining advanced-moderate;

^d^One of them had lesions disappearance at this time.

The results of the evaluations are shown in Table 3. The overall response, which was the main study outcome, was significantly better in those patients treated with the combination (72% vs. 36%). All responses were complete, except for one patient in the IFN group who responded partially after treatment but reached complete response at the end of follow-up. The difference persisted during the follow-up (67% vs.29%). These analyses were done under the intention-to-treat principle, where all withdrawals are considered as failures. If the analysis is made with each patient's last evaluation differences are larger and more significant.

The clinical, radiological, and bacteriological assessments taken independently were also better for the IFN gamma group. Improvement was obtained for the presence of respiratory symptoms such as dyspnea only in the IFN group. Additionally, withdrawals in this group were also asymptomatic, which only happened for one of the placebos. BMI increments were also larger among IFN treated patients. Regarding clinical general status and its improvement, statically significant differences were achieved. Importantly, 7/14 patients from the IFN group but none among the controls improved their general status (passed from "bad" to "moderate" or from "moderate" to "good") just one month after the therapy onset. Among survivors, there were two patients in the placebo group who worsened their general clinical condition. The only patient where this happened in the IFN group was infected with *M. fortuitum-cheloneae*.

**Figure 1:**
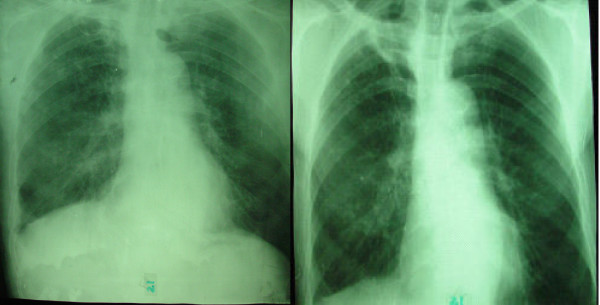
Radiological improvement with IFN gamma treatment as adjuvant to chemotherapy. (A): Advanced fibrosis and cavitations, sited on vertex and parahilar areas, and (B) complete resolution of the lesions after treatment and follow-up.

All patients, except one in the IFN group, presented fibrotic lesions. Twelve individuals in each group had cavitations. Pleural thickness and interstitial and/or alveolar infiltrate were seen in 15 and 13 subjects, respectively. Apical and parahilar lesion localization were most common. Advanced lesions predominated at entry in both groups. At the end of treatment more patients treated with IFN gamma reduced lesion extension: advanced lesions persisted in only 3 patients from this group vs. more than half in the placebo group. A 61 years-old male, 2 years with the disease, which initially had advanced lesions evidenced a total disappearance of lesions after IFN treatment and follow-up (Figure 1). The other 12 patients in the IFN group who where evaluated at month 18 improved their radiological condition, 5 of them reached the minimum lesion extension and 4 up to moderate. On the contrary, among the controls, 6 improved, but only one reached minimum extension, and it was a subject bearing *M. kansasii *infection. Additionally, one patient in this group worsened his status at 18 months follow-up. Cavitary lesions disappeared in 5 patients in the IFN group (42%), at an average of 6 months (range: 1 – 12 months), but only in the *M. kansassi *bearing patient in the placebo group (8%), at 5 months.

Bacteriological response by direct examination and culture occurred in both groups at the end of treatment. However, during follow-up there were more relapses in the placebo group (3 vs. 1 in the experimental group) and besides, there was one late positive sputum conversion to negative among the IFN receiving patients. Statistically significant differences were attained regarding sputum culture codification at the end of follow-up.

**Figure 2:**
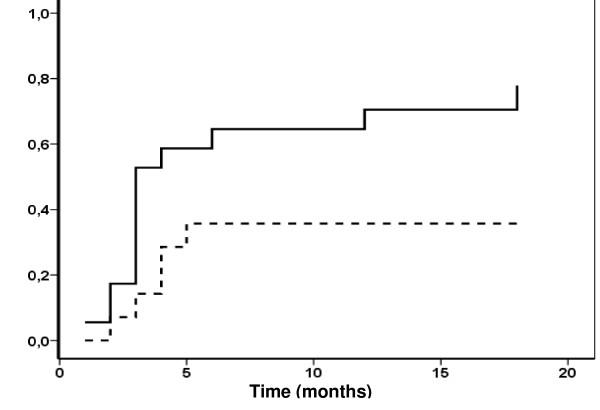
Kaplan-Meier estimates of probability of overall complete response in patients with pulmonary atypical Mycobacteriosis treated with IFN gamma (solid line) or placebo (dashed line) adjuvant to chemotherapy.

Overall complete response was obtained earlier in patients treated with the combination (Figure 2; log rank test, p = 0.024). Interestingly, the only complete responder at 1st month of treatment corresponded to the IFN group. All symptoms disappearance, or particularly dyspnea, and radiological improvement behaved the same (graphs not shown). The most important differences in improvement rate concerned the radiological evaluation. After one month of treatment, 4 patients in the IFN group but none among placebos showed pulmonary lesions improvement. At this point, 60% of patients with initial dyspnea in the IFN group, no longer presented this symptom, in contrast to16.7% in the other group.

**Figure 3:**
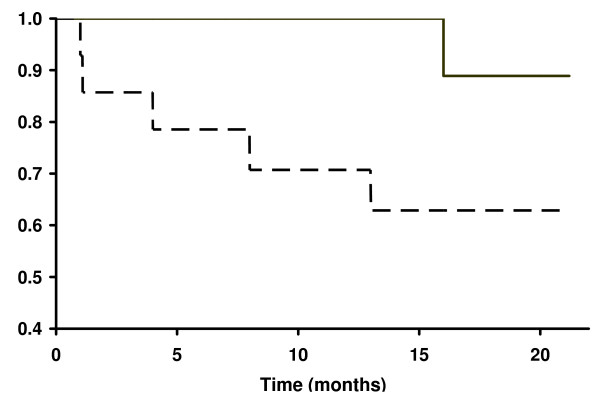
Kaplan-Meier estimates of probability of overall survival among patients with pulmonary atypical Mycobacteriosis treated with IFN gamma (solid line) or placebo (dashed line) adjuvant to chemotherapy.

Survival profiles are shown in Figure 3. During the entire study, 5 patients (35.7%) deceased in the control group (median survival: 4.5 months) and only 2 (11.1%) in the IFN group. Both deaths in the IFN group occurred at 16 months, as complete responders. Among the control patients, 4 died as non-responders and the other one as partial responder.

Oxidative stress parameters basal mean values were importantly increased in the patients as compared to a group of 60 Cuban healthy individuals [26] (Table 4). Significant decrements in SOD and AOPP values were detected in the IFN group (Table 5). The difference was more pronounced for AOPP, since it decreased in 9/10 IFN treated patients whereas increased in 5/7 from the placebo group. For the other parameters important changes were not perceived.

**Table 4:** Comparison of oxidative stress parameters between patients with pulmonary atypical Mycobacteriosis and healthy individuals.

**Parameter**	**Patients N = 18**	**Healthy subjects N = 60 (from ref. 27)**
Catalase (U/mL/min)	457 ± 286	162 ± 23
SOD (U/mL/min)	32.4 ± 15.7	1.5 ± 0.1
Catalase/SOD	0.02 ± 0.02	0.11± 0.20
AOPP (μM)	70.1 ± 30.2	12.1 ± 0.9
MDA (μM)	16.8 ± 9.8	1.8 ± 0.1
PLPP (μM)	37.1 ± 20.0	7.6 ± 1.3
THP (μM)	181.7 ± 69.6	103.8 ± 17.7

Data are expressed as mean ± SD.

Regarding gene modulation, the most important variations were detected within the groups after treatment (Table 5). TGF beta increased significantly in the placebo group, whereas MCP-1 decreased in the IFN gamma group. TNF alpha values, although slightly different before treatment, increased significantly in the placebo group, while did not change with IFN therapy. No changes were detected for the other genes evaluated (results not shown).

**Table 5:** Molecular expression and oxidative stress studies.

**Parameter**	**Month**	**IFN gamma**	**Placebo**	**p (Student's t)**
**Oxidative stress**		**N = 10**	**N = 8**	
SOD (U/mL/min)	0	36.2 ± 17.8	27.2 ± 11.5	0.23
	6	28.6 ± 12.6	29.8 ± 16.5	0.85
	P (6 *vs *0)	0.034	0.612	
AOPP (μM)	0	71.3 ± 35.2	60.8 ± 34.5	0.46
	6	48.4 ± 15.2	65.7 ± 27.6	0.12
	P (6 *vs *0)	0.008	0.734	

**Genetic modulation (relative to GAPDH × 100)**		**N = 8**	**N = 6**	
TGF beta	0	113 ± 37	113 ± 29	0.99
	6	152 ± 106	235 ± 232	0.80
	p (6 *vs *0)	0.401	0.028	
TNF alpha	0	37 ± 35	68 ± 21	0.080
	6	37 ± 34	81 ± 28	0.025
	p (6 *vs *0)	0.946	0.370	
MCP-1	0	112 ± 58	38 ± 29	0.015
	6	59 ± 27	69 ± 39	0.60
	p (6 *vs *0)	0.032	0.244	

Data are expressed as mean ± SD.

Analyses within groups were performed by the Student's paired t test.

Seventeen adverse events were presented during the treatment (Table 6). At least one event occurred in 66.7% of the patients in the IFN group and 42.9% in the placebo group. Arthralgias (50.0%), fever (44.4%), chills (33.3%), anorexia (27.8%) and asthenia (22.2%) prevailed in the IFN group, where a higher frequency was detected for fever and chills, which were not present in the placebo group. Most of the events in the IFN group were classified as moderate (60%), none of them severe. All of them were well controlled with or without treatment. Respiratory or cardiorespiratory manifestations, closely related to the disease, were not accounted as adverse reactions.

Significant differences or variations in vital signs and clinical laboratory tests were not detected. Only a moderate creatinine increase and a mild anemia in a single patient of the IFN group were considered as adverse reactions. On the contrary, in this group, 3 patients with complete response normalized their GSR values, 2 of them with initial values > 100 mm/h. A mean reduction of 22 mm/h (71 to 49 mm/h) took place in the IFN group, while in the placebo group GSR values remained stable.

**Table 6:** Adverse events during treatment.

**Adverse reaction**	**IFN gamma N = 18**	**Placebo N = 14**
Any adverse event	12 (66.7%)	6 (42.9%)
Arthralgias	9 (50%)	2 (14.3%)
Fever	8 (44.4%)	0
Chills	6 (33.3%)	0
Anorexia	5 (27.8%)	2 (14.3%)
Asthenia	4 (22.2%)	0
Headache	3 (16.7%)	0
Diarrhea	2 (11.1%)	1 (7.1%)
Pruritus	2 (11,1%)	1 (7.1%)
Epigastralgia	1 (5.6%)	1 (7.1%)
Myalgias	0	1 (7.1%)
Vomiting	1 (5.6%)	0
Depression	0	1 (7.1%)
Anemia	1 (5.6%)	0
Pain at the injection site	1 (5.6%)	0
Increase of creatinine	1 (5.6%)	0
Limb edema	0	1 (7.1%)
Nausea	0	1 (7.1%)

## Discussion

This report constitutes the first and largest randomized, controlled clinical study, using an immunomodulating agent systemically in atypical Mycobacteria infection. The group of patients treated with IFN gamma and antibiotics had a better response from the clinical, radiological and bacteriological points of view. Response rate in the IFN group doubled that from the controls after 6 months of treatment and the difference persisted after one year follow-up. Patients that received IFN gamma responded 5 to 6 months before those with placebo, with respect to all evaluation criteria. The earlier disappearance of dyspnea in the IFN group was remarkable since this symptom affects quality of life more than others. This was not only statistically but also clinically significant. There was also an advantage in overall survival. In fact, except for one case, all patients treated with IFN gamma received benefit from treatment. The 66.7% response rate was calculated under intention-to-treat basis, considering the abandoners and deceased as failures. But in this group one of the abandoners had complete response at withdrawal after the first month of treatment and the two deaths were due to myocardial infarcts. Both deceased cases had had complete response at the end of treatment. In the control group one of the abandoners had responded but four of the five deceased were non responders.

Advanced age and male gender predominated in this study, comparable to reported [1]. However, antecedents of respiratory tract affectations were scarce. As expected, MAC species predominated. *M. fortuitum-cheloneae *was present in one patient of the IFN group, which was the only one that did not receive benefits from treatment. Coincidently, infection by this species has been reported in patients with anti-IFN gamma autoantibodies [27]. Nevertheless, infection with this specie is less common. The other species, *M. kansassi*, presented in one patient with placebo, is more sensitive to first line therapy. This patient was the best responder in this group; the only one where cavitary lesions disappeared. Obviously, differences between groups could be more significant if no-MAC species are excluded from analysis, but this was not previewed in the protocol.

The antibiotic scheme used in this trial was in correspondence with the best update recommended. Based on *in vivo *and *in vitro *studies treatment should include: (i) a macrolide (clarithromycin or azithromycin), (ii) ethambutol, (iii) rifampin or rifabutin, and (iv) ciprofloxacin, streptomycin or clofazimine. However, the response level obtained in the IFN group is rarely achieved with antibiotics only. Besides, clinical studies are not controlled, often use more flexible response criteria and longer periods of treatment [28].

Radiological response during treatment was the most significant benefit added by IFN gamma. Improvement was evident since the first month and there was even complete lesion disappearance in one patient, which has not been reported after such a relatively short follow-up time. This lesion extension reduction had consequences on the patients' clinical performance with dyspnea and other symptoms reduction or disappearance. In this case, radiology had a higher clinical impact than bacteriology. Several patients in the placebo group even with sputum conversion, never experienced radiological improvement, most of them worsened lesions and had bacteriological relapse. Contrarily, in the IFN group, some patients with sputum-culture positive had an early lesions reduction, with symptoms disappearance and had later complete response. Improvement difference included not only cavitary lesions reduction (42% vs. 8%) but also fibrosis and infiltrate extension. This suggests a different mechanism for the additional radiological improvement, probably related to the potent antifibrotic effect of IFN gamma, whether the *Mycobacterium *is present or not.

Treatment was well tolerated. Flu-like symptoms such as fever, chills and arthralgias are among those expected for interferons since their first clinical applications [29]. Reduction in blood cell counts and moderate creatinine increase, which appeared in one case each have been also reported. Gastrointestinal events (vomiting, diarrheas) are probably more related to the use of the antibiotics [1,5,7]. All deaths in the IFN group were during the follow-up period so they are unlikely related to treatment. However deaths can be related direct or indirectly to the disease since it is a predisposing factor for lung cancer, pulmonary thromboembolism, and myocardial infarction, which are all preceded by cardiorespiratory events.

The role of IFN gamma as the main macrophage – activator Th1 cytokine has been clearly established in animal models infected with *M. avium *[30,31]. IFN gamma action on the macrophages leads to intracellular *Mycobacteria *killing. It stimulates macrophages to produce TNF alpha, oxygen free radicals and nitric oxide, increases MHC surface antigens and Fc receptors display, decreases lysosomal pH, and increases the intracellular concentration of some antibiotics (e.g. macrolides) [9,30]. Therefore, its use as adjuvant is justified since existent multidrug therapy, despite its limited efficacy, must be offered to the patients.

Regarding its antifibrotic effect, IFN gamma inhibits lung fibroblast proliferation and chemotaxis in a dose dependent manner and reduces collagen synthesis [14]. Furthermore, this protein is a potent antagonist of TGF beta [15], involved directly in severe lung fibrosis progression [32]. IFN gamma contributes to the tissue repair and its remodeling [33]. These actions were also evident in the results reported with IFN gamma in patients with idiopathic pulmonary fibrosis [34,35] and suggests that this cytokine could have future indications in other pulmonary diseases where fibrosis is present, although a definite effect on survival is still to be demonstrated.

Systemic or aerosolized IFN gamma have been reported as satisfactory in other similar intracellular infections [36-41], but none of these studies was controlled. The concept that IFN gamma can be useful in these atypical Mycobacterial infections is supported by several reports on individuals where the IFN gamma action is impaired. Mutations in the IFN gamma receptor genes are associated with frequent recurrences or the most serious forms of the disease [11-13]. Patients with defects in the production of IFN gamma or other cytokines or partial deficiencies of IFN gamma receptor can obtain benefits with IFN gamma treatment [10,13,42]. Additionally, patients without genetic disorders but with serum anti-IFN gamma autoantibodies have a higher susceptibility to develop Mycobacteriosis [43].

IFN gamma has been effective as adjuvant in AIDS patients co-infected with MAC, where a clear decrement in the bacteremia was verified. These results were obtained in patients with low CD4+ lymphocytes counts, suggesting a non T cell-mediated effect [16]. Fifteen patients with disseminated MAC and other nontuberculous mycobacteria infections were treated subcutaneously during one year or more, 13 of them improved and 7 had even apparent disease eradication [20]. Other groups of HIV negative immunocompromised patients infected with *M. avium *received clinical and bacteriological benefits of this treatment as well [17,18]. Short courses of the cytokine administered by alveolar route seemed to be effective in pulmonary MAC, since their use in one patient [19]. However, it has been recently reported that a randomized trial testing this option was stopped early due to lack of efficacy [44]. Since different routes of administration (aerosols, subcutaneous, intramuscular) have been used, this aspect should be further investigated.

Oxidative stress indicates an excessive production of oxygen reactive species (ROS), together with an inadequate antioxidant defense [45]. Scarce information exists about pulmonary infections including *M. avium *in this sense. The redox unbalance was evidenced through oxidative damage markers increments, as shown by the comparison with healthy control reference values. AOPP are considered as the major free radicals-dependant protein damage indicator [46]. As result of the exposition to radicals, proteins could suffer modifications, fragmentation, aggregation, changes on the absorption, decrease or loss of their biological function or proteolysis [47]. After treatment, a significant reduction was detected in the IFN group. Their decrease or return to normal values can be secondary to infection eradication. In the same group, a SOD reduction was also observed. Although SOD plays a role in the defense against toxic oxygen metabolites, in most of the cases its presence indicates tissue damage and participates in reactions that drive to formation of hydrogen peroxide and iron, producers in turn of new toxic molecules [48]. Therefore, their decrease can be taken as another favorable presage.

Genetic modulation data obtained are consistent with the clinical results. A significant increment in TGF beta was obtained in the placebo group. This factor counteracts T-cell proliferation and the production of IFN gamma, TNF alpha and pro-inflammatory chemokines [49], in addition to its pro-fibrotic activity [33], and thus helps *Mycobacteria *to avoid immune reactions and survive. MCP-1 participates in leukocyte recruitment toward granuloma [50]. It also inhibits IL-12 production favoring infection [51]. Therefore, MCP-1 decrease in the IFN group could be associated to pathogen control leading to granuloma resolution. The same interpretation is possible for the TNF alpha increase that occurred in the placebo group but not in the IFN treated patients. Although IFN gamma has been considered as a pro-inflammatory molecule, more and more evidences of anti-inflammatory actions exist, which supposes a dual effect [52].

## Conclusion

The results obtained may justify the rationality to use IFN gamma as adjuvant to antimycobacterial drugs in patients with pulmonary atypical Mycobacteriosis, mainly in MAC infection, which is the most frequent. Their combination could reduce treatment duration, toxicities and possible relapses. In some cases it could prevent recessional surgery. Further, more extensive, controlled clinical trials are encouraged to confirm this assessment.

## Abbreviations

AOPP: Advanced oxidation protein products; BMI: Body mass index; BMRC: British Medical Research Council; CCR: Chemokine (CC motif) receptor; MAC:*Mycobacterium avium *complex; MHC: Major histocompatibility complex; GAPDH: Glyceraldehyde-3-phosphate dehydrogenase; GSR: Globular sedimentation rate; HIV: Human Immunodeficiency Virus; IFN: Interferon; IL: Interleukin; MDA: Malonyldialdehyde; MCP: Monocyte chemoattractant protein; PBMN: Peripheral blood mononuclear; PLPP: Potential lipid peroxidation products; ROS: Oxygen reactive species; SOD: Superoxide dismutase; STAT: Signal transducer and activator of transcription; TGF: Transforming growth factor; TNF: Tumor necrosis factor; T-bet: Transcription factor associated to Th1 lymphocytes; THP: Total hydroperoxides.

## Competing interests

Authors IGG, CMVS, TRG, IBR, LGM and PALS are employees of the Center for Biological Research, which is part of the Center for Genetic Engineering and Biotechnology, Havana network, where human recombinant IFN gamma is produced. The rest of the authors have no competing interests at all.

## Authors' contributions

MTMV conceived the study and was the main medical investigator. IGG participated in the study design, coordination and monitoring, and wrote the manuscript draft. YSH, MVQ, GJM, and NFO took care of patient recruitment, management, and follow-up. CMVS participated in the study design and results analyses. TIRG and IBR carried out molecular biology studies. RBSO did the radiological evaluations and CRA made the bacteriological determinations. LGM contributed as study monitor. GMS developed the oxidative stress evaluations. PALS took part in the design, results analyses and manuscript writing. All authors read and approved the final manuscript.

## Appendix

The other members of the MACGAM Study Group are: Roberto Suárez-Méndez, Dalia Carbonell-Freire (†), Nancy Silva-Sánchez, Delfina Machado-Molina, Eloína Turró-Soto, Helia I Herrera-García, and Madelyn Martínez-Soret from the "Benéfico Jurídico" Hospital, Havana, Mildrey Iglesias, "Amalia Simoni" Hospital, Camagüey, Elizeth García-Iglesias and Orlando C San Jorge-Mesa, Center for Biological Research, Havana.

## Pre-publication history

The pre-publication history for this paper can be accessed here:


